# Vitamin D Deficiency in Preclinical Medical Students: Neuroendocrine, Cognitive, and Immune Effects

**DOI:** 10.7759/cureus.115944

**Published:** 2026-09-08

**Authors:** Leah Degard-Edmond, Raju Panta

**Affiliations:** 1 Osteopathic Medicine, Burrell College of Health Sciences School of Osteopathic Medicine, Melbourne, USA; 2 Physiology and Pathology, Burrell College of Health Sciences School of Osteopathic Medicine, Melbourne, USA

**Keywords:** academic performance, cognitive function, hypothalamic-pituitary-adrenal axis, immune function, neuroendocrine regulation, neuroinflammation, preclinical medical students, stress resilience, vitamin d deficiency, vitamin d supplementation

## Abstract

Vitamin D is increasingly recognized as a multifunctional hormone that extends beyond its traditional role in skeletal health to influence neuroendocrine regulation, cognitive performance, and immune function. Preclinical medical students represent a population at particularly high risk for vitamin D deficiency because of prolonged indoor study, limited sunlight exposure, sedentary lifestyles, and demanding academic schedules. This narrative review examines current evidence regarding vitamin D metabolism and its effects on the hypothalamic-pituitary-adrenal axis, stress resilience, cognition, neuroprotection, and immune homeostasis, with specific relevance to preclinical medical education. Available studies consistently demonstrate a high prevalence of vitamin D deficiency among medical students worldwide, with rates frequently exceeding 70%. Emerging evidence suggests that inadequate vitamin D status may contribute to impaired stress adaptation, mood disturbances, neuroinflammation, reduced cognitive performance, and immune dysregulation. However, the evidence is limited by the predominance of observational and cross-sectional studies, variability in definitions of vitamin D sufficiency, heterogeneous study populations, and a lack of prospective investigations evaluating causality. Furthermore, data from U.S. medical student populations are limited. Despite these limitations, optimization of vitamin D status through lifestyle modification, dietary measures, and appropriate supplementation may support student well-being and academic success. Further research is needed to clarify prevalence, determine optimal vitamin D targets for neurocognitive health, and evaluate the effectiveness of preventive and therapeutic interventions in medical trainees.

## Introduction and background

Vitamin D is a fat-soluble vitamin synthesized primarily in the skin following exposure to ultraviolet B (UVB) radiation and is available in two major forms: ergocalciferol (vitamin D₂) and cholecalciferol (vitamin D₃) [1]. Beyond its well-established role in calcium and bone metabolism, vitamin D functions as an important regulator of neuroendocrine, immune, and metabolic processes. Vitamin D deficiency has been associated with altered brain function and an increased risk of chronic diseases, including hypertension, type 2 diabetes, autoimmune disorders, depression, anxiety, and certain cancers, potentially through effects on inflammation, immune regulation, and metabolic homeostasis [2].

The widespread expression of vitamin D receptors (VDRs) in immune cells, including B cells, T cells, and antigen-presenting cells, highlights the importance of vitamin D in maintaining immune balance and controlling inflammatory responses [3]. Vitamin D is also increasingly recognized as a neuroactive hormone, with VDRs and vitamin D-metabolizing enzymes present in several brain regions, including the hypothalamus, where they influence neuroendocrine signaling and physiological homeostasis [4]. Emerging evidence suggests that low vitamin D levels are associated with depression, anxiety, and other adverse mental health outcomes through mechanisms involving neuroinflammation, oxidative stress, and altered neurotransmission [4,5].

Vitamin D deficiency is particularly common among university students and has been linked to increased psychological stress, anxiety, and depressive symptoms [6,7]. Preclinical medical students may be especially vulnerable because of demanding academic schedules, prolonged indoor study, and limited sunlight exposure. Studies from medical student populations worldwide have reported alarmingly high rates of vitamin D deficiency. For example, a study among Saudi medical students found deficiency in 96% of participants, with significantly lower vitamin D levels among female students [8].

Vitamin D deficiency may influence key domains emphasized in contemporary medical student well‑being frameworks, including cognitive performance, stress regulation, and overall physiological resilience, suggesting that it represents a modifiable factor relevant to academic success and burnout prevention. Incorporating vitamin D awareness into wellness programming and curricular guidance may complement existing institutional efforts to support learning efficiency, emotional stability, and student engagement during preclinical training.

This narrative review examines current evidence regarding vitamin D deficiency among preclinical medical students, with a focus on its neuroendocrine, cognitive, and immune implications and their potential impact on academic performance and long‑term health outcomes.

## Review

Methodology

This study was conducted as a narrative review of the literature on vitamin D deficiency among preclinical medical students and its associations with neuroendocrine function, cognition, and immune regulation. A narrative approach was selected because the existing evidence base is highly heterogeneous, spanning diverse study designs, variable outcome measures, and mechanistic investigations that are not amenable to systematic review methodology. A literature search was performed using PubMed/MEDLINE, Scopus, Google Scholar, and the Cochrane Library for articles published between January 2012 and July 2026. Search keywords and medical subject headings included vitamin D deficiency, 25-hydroxyvitamin D, medical students, preclinical students, cognition, academic performance, HPA axis, hypothalamus, and immune function.

Peer-reviewed English-language studies evaluating vitamin D status in medical students or similar university populations were included. Studies reporting serum 25-hydroxyvitamin D levels and outcomes related to cognitive performance, psychological stress, neuroendocrine regulation, or immune function were considered. Non-English articles, case reports, and studies lacking clinical relevance were excluded.

Titles, abstracts, and full texts were screened for relevance. Data on study design, population characteristics, vitamin D status, prevalence of deficiency, and relevant health outcomes were extracted and synthesized qualitatively. Findings were organized into thematic categories including neuroendocrine regulation, cognitive outcomes, immune function, prevalence, and risk factors of vitamin D deficiency among medical students. Because this review used previously published data, institutional review board approval and informed consent were not required.

Vitamin D synthesis, activation, and molecular action

Vitamin D is a secosteroid hormone that plays a fundamental role in maintaining human health through its effects on metabolic, endocrine, and immune processes. Beyond its classical function in calcium and phosphate regulation, vitamin D influences the expression of numerous genes involved in hormonal signaling, immune function, and cellular homeostasis, reflecting its broad physiological importance [9].

Vitamin D is acquired through both dietary intake and endogenous synthesis in the skin. Dietary sources provide vitamin D₂ (ergocalciferol), derived primarily from plants and fungi, and vitamin D₃ (cholecalciferol), obtained from animal-based foods and produced in the skin following exposure to UVB radiation. During cutaneous synthesis, UVB radiation converts 7-dehydrocholesterol in the epidermis to previtamin D₃, which undergoes thermal isomerization to form vitamin D₃. The newly synthesized vitamin D₃ enters the circulation bound to vitamin D-binding protein and is transported to the liver for further metabolism [9-11].

In the liver, vitamin D is hydroxylated to 25-hydroxyvitamin D [25(OH)D], the major circulating metabolite and the most widely used indicator of vitamin D status. Subsequently, 25(OH)D undergoes a second hydroxylation primarily in the kidneys to form 1,25-dihydroxyvitamin D [1,25(OH)₂D], or calcitriol, the biologically active form of vitamin D [10,11]. Calcitriol exerts its physiological effects by binding to the VDR, a nuclear transcription factor expressed in numerous tissues. Upon activation, the VDR forms a heterodimer with the retinoid X receptor (RXR), and the resulting complex binds to vitamin D response elements within target genes, regulating the transcription of hundreds of genes involved in mineral metabolism, cellular differentiation, immune regulation, and metabolic homeostasis [9,10].

Although traditionally recognized for its role in calcium and phosphate homeostasis, vitamin D is now understood to have pleiotropic effects extending beyond skeletal health. The widespread distribution of VDR and vitamin D-metabolizing enzymes in immune, endocrine, and neural tissues supports its involvement in diverse physiological processes. Notably, the presence of VDR in several brain regions, including the hypothalamus, suggests a role in neuroendocrine signaling, energy balance, and the maintenance of systemic homeostasis [4]. Furthermore, vitamin D signaling has been implicated in neuroprotection, modulation of neuroinflammation, and regulation of neurotransmitter pathways, highlighting its potential relevance to cognitive function and mental health [9].

Vitamin D and the hypothalamic-pituitary-adrenal axis

Beyond its established role in mineral metabolism, vitamin D has important functions within the central nervous system. Evidence indicates that vitamin D participates in neurodevelopment, neurotransmitter regulation, and neurotrophic signaling, including pathways involved in dopaminergic activity and neuronal survival. Through these mechanisms, vitamin D contributes to brain plasticity, cognitive function, and overall neurological health. Recent studies have also linked vitamin D deficiency to impaired brain function and an increased risk of neuropsychiatric and neurodegenerative disorders, although the clinical benefits of vitamin D supplementation on neurological outcomes remain inconclusive [12-14].

Vitamin D is increasingly recognized as a modulator of neuroendocrine function, particularly through its interactions with the hypothalamic-pituitary-adrenal (HPA) axis. The presence of VDRs and vitamin D-metabolizing enzymes in the hypothalamus, pituitary gland, and other brain regions suggests that vitamin D signaling may influence neuroendocrine pathways involved in stress adaptation and homeostatic regulation [4,15]. Following activation to calcitriol, vitamin D binds to the VDR and regulates the transcription of numerous genes involved in cellular signaling, inflammation, and endocrine function, thereby contributing to the maintenance of physiological balance [4].

Further evidence suggests that vitamin D may influence HPA axis activity through its anti-inflammatory and neuroprotective actions. Because chronic activation of the HPA axis is associated with elevated cortisol levels, impaired cognition, and adverse psychological outcomes, disruptions in vitamin D signaling may contribute to altered stress responses and reduced resilience [5,15]. These mechanisms may be particularly relevant in preclinical medical students, who frequently encounter substantial academic stress and may be vulnerable to both vitamin D deficiency and stress-related impairments in well-being and cognitive performance.

Vitamin D deficiency, cortisol regulation, and stress resilience

Vitamin D deficiency may reduce stress resilience by impairing VDR-mediated neuroprotective signaling within key stress-responsive brain regions, including the hypothalamus and prefrontal cortex [16]. Calcitriol exerts neurobiological effects by crossing the blood-brain barrier and binding to nuclear VDRs, where it forms heterodimers with RXRs to regulate the expression of genes involved in neuronal survival, neuroplasticity, and cellular homeostasis [17,18]. This signaling pathway plays an important role in maintaining emotional and cognitive stability.

Evidence further indicates that vitamin D participates in an intrinsic brain auto/paracrine system. Vitamin D metabolites can enter the central nervous system and undergo local activation within glial and neuronal cells, allowing region-specific regulation of neurophysiological processes [18,19]. The widespread distribution of VDRs and vitamin D-metabolizing enzymes throughout the brain, including the hypothalamus and other regions involved in cognition and behavior, underscores the importance of vitamin D signaling in neuroendocrine homeostasis [20].

The HPA axis is central to the physiological stress response and cortisol regulation. Emerging evidence suggests that vitamin D influences HPA axis activity by modulating cortisol-related signaling pathways and downstream neuroendocrine responses [16]. There is a significant association between serum vitamin D concentrations and HPA axis hormone levels in patients with post-traumatic stress disorder, suggesting that inadequate vitamin D status may contribute to altered stress responses. Experimental studies further demonstrate that vitamin D₃ administration attenuates stress-induced biochemical disturbances and prevents memory deficits under conditions of chronic unpredictable stress, supporting a protective role for vitamin D against stress-related neurocognitive dysfunction [16,18,21].

Inflammation and oxidative stress represent additional mechanisms linking vitamin D deficiency with reduced psychological well-being. Lower serum 25(OH)D concentrations have been associated with increased symptoms of anxiety, depression, psychosocial stress, and poorer overall mental health among young adults [22]. Vitamin D’s antioxidant and anti-inflammatory properties may contribute to neuronal protection by promoting neurogenesis, synaptic plasticity, and cellular resilience, thereby supporting the maintenance of normal brain function [23].

Clinical evidence also suggests that vitamin D status may influence cognitive performance during stressful conditions. A randomized controlled trial demonstrated that vitamin D status affected planning and problem-solving performance under stress exposure, indicating a potential role for vitamin D in cognitive resilience [19]. These findings are particularly relevant for preclinical medical students, who frequently encounter sustained academic stress and high cognitive demands.

Beyond its role in stress regulation, vitamin D functions as a neuroactive steroid that supports neurotransmission, neuroplasticity, and neuronal survival [17,18]. The presence of VDRs and vitamin D-metabolizing enzymes in brain regions such as the hippocampus, amygdala, and prefrontal cortex further highlights its importance in maintaining executive function, emotional regulation, and adaptive behavioral responses [17,18,20]. Consequently, vitamin D deficiency may increase vulnerability to stress-related cognitive and psychological impairments, making adequate vitamin D status a potentially important factor in promoting resilience and optimizing performance among medical students.

Vitamin D and cognitive performance

Vitamin D has emerged as an important neuroactive hormone with significant effects on cognitive function and brain health. Its biologically active form, 1,25-dihydroxyvitamin D₃, acts through VDRs and vitamin D-metabolizing enzymes that are widely expressed throughout the central nervous system, including regions involved in learning, memory, executive function, and behavioral regulation [4,24,25]. Through VDR signaling in brain regions, vitamin D supports neuronal development, neuroprotection, synaptic plasticity, neurotransmission, and gene expression pathways that are essential for optimal cognitive performance [25,26].

Cognitive impairment has been linked to disruptions in intracellular calcium homeostasis, a process that is critical for neuronal excitability, synaptic transmission, neuroplasticity, and memory formation. Dysregulated calcium signaling can impair neuronal and synaptic function, alter neurotransmitter release, and contribute to deficits in learning and memory. Furthermore, abnormalities in calcium-dependent signaling pathways may influence the expression and activity of neurotrophic factors that support neuronal survival, synaptic plasticity, and adaptive cognitive processes, ultimately compromising memory consolidation and cognitive flexibility [27,28].

Given the important role of vitamin D in regulating HPA axis function and maintaining neuroendocrine homeostasis, persistent vitamin D deficiency may impair physiological pathways that support cognitive adaptation, stress regulation, and behavioral functioning [29,30]. Emerging evidence suggests that inadequate UVB exposure and subsequent reductions in vitamin D availability may contribute to alterations in neurobiological pathways involved in mood regulation and psychiatric disorders. These observations support a broader role of vitamin D in brain health and psychological resilience beyond its traditional functions in skeletal metabolism [31]. Increasing evidence supports an interconnected relationship between neurodevelopment, cognitive function, and bone metabolism [32]. Disruptions in biological pathways linking these systems may contribute to neurodevelopmental and cognitive impairments, suggesting that deficiencies in factors critical for both brain and skeletal health, such as vitamin D, could have broader consequences for learning, memory, and overall neurological function [29-32]. In demanding academic environments such as medical education, these effects may hinder the acquisition and consolidation of complex knowledge and contribute to poorer cognitive and academic outcomes.

Vitamin D and immune function

Vitamin D deficiency is associated not only with impaired calcium and phosphate homeostasis and reduced bone health but also with significant alterations in immune regulation. Emerging evidence indicates that inadequate vitamin D status contributes to immune dysregulation, resulting in enhanced inflammatory activity, impaired immune tolerance, and an increased risk of autoimmune diseases [3,7,33]. Furthermore, vitamin D deficiency has been linked to greater susceptibility to infections and disturbances in hematopoietic and immune cell function [33]. These observations are supported by studies demonstrating that vitamin D modulates both innate and adaptive immune responses through its actions on immune cells expressing VDRs and vitamin D-metabolizing enzymes, thereby promoting immune homeostasis and reducing excessive inflammatory responses [33,34].

Vitamin D functions as a critical immunomodulatory hormone that regulates both innate and adaptive immune responses through VDRs expressed on monocytes, macrophages, dendritic cells, and lymphocytes. By modulating immune cell differentiation, cytokine production, and inflammatory signaling pathways, vitamin D helps maintain immune tolerance while limiting excessive inflammatory responses that may be exacerbated by chronic psychological stress [7,35,36]. Furthermore, vitamin D enhances host defense mechanisms by stimulating the production of antimicrobial peptides and strengthening innate immune functions, thereby improving resistance to infectious agents and reducing the risk of chronic inflammatory and autoimmune conditions. Collectively, these actions support immune homeostasis and may help protect against systemic inflammation and immune-mediated tissue injury [34-36].

Vitamin D signaling may mitigate chronic neuroinflammation through its immunomodulatory and neuroprotective actions, including the regulation of inflammatory cytokine production and the suppression of excessive glial cell activation [17,37]. Through these mechanisms, vitamin D helps maintain neuronal homeostasis, reduce oxidative and inflammatory damage, and preserve neural integrity [38]. Persistent vitamin D deficiency has been associated with enhanced neuroinflammatory activity, which may contribute to adverse neurological outcomes and increase susceptibility to neurodegenerative and neuropsychiatric disorders [17,37,38].

Defining adequate vitamin D levels

Determining an optimal threshold for vitamin D sufficiency remains an area of ongoing scientific discussion. A major challenge stems from differences in laboratory assays, measurement techniques, and the lack of universal standardization for circulating 25(OH)D concentrations, which can lead to variability in reported values and inconsistencies in the clinical interpretation of vitamin D status [39,40]. Despite substantial advances in assay standardization, disagreements persist regarding the serum 25(OH)D concentrations required to support various health outcomes, reflecting the complexity of establishing universally accepted diagnostic criteria for vitamin D sufficiency [39-41].

The optimal serum concentration of 25(OH)D and the corresponding daily vitamin D intake required to achieve it remain subjects of ongoing debate. Although clinical guidelines generally recommend maintaining sufficient vitamin D status through dietary intake, supplementation, and sun exposure, recommendations vary among professional organizations because of differences in the health outcomes used to define adequacy and the substantial interindividual variability in vitamin D requirements [42-44]. The Endocrine Society guideline recommends higher intake levels for individuals at risk of deficiency and identifies 4,000 IU/day as the tolerable upper intake level for adults, while acknowledging that higher doses, up to 10,000 IU/day, may be used safely under specific clinical circumstances and medical supervision [43]. Contemporary evidence further suggests that vitamin D intakes above traditional minimum recommendations may be necessary for some individuals to achieve and maintain optimal serum 25(OH)D concentrations and support broader health outcomes beyond skeletal health [42,44].

However, increasing evidence suggests that serum 25(OH)D concentrations sufficient for skeletal health may not necessarily be optimal for neurological function. Recent studies indicate that vitamin D plays important roles in neuroprotection, synaptic plasticity, neuroinflammation regulation, and cognitive maintenance, raising the possibility that higher circulating vitamin D concentrations may be required to support brain health than those traditionally recommended for bone metabolism alone [25]. Despite these observations, translating higher vitamin D targets into clinical practice remains challenging because randomized controlled trials and meta-analyses have reported heterogeneous outcomes, and a clear dose-response relationship between vitamin D supplementation and improvements in depressive symptoms or cognitive performance has not yet been established [45]. Furthermore, emerging evidence suggests that vitamin D may influence neurobiological function through epigenetic mechanisms, including the regulation of gene transcription and chromatin remodeling pathways, which may affect neuronal resilience and stress responsiveness [46-47]. Consequently, although serum 25(OH)D concentrations above 30 ng/mL are widely accepted as indicative of sufficiency, the optimal level required to maximize cognitive and neuropsychiatric health remains an area of ongoing investigation [17,45-47].

Recent findings suggest that circulating 25(OH)D concentrations may not fully represent vitamin D activity within the central nervous system. Experimental evidence indicates that vitamin D deficiency is associated with behavioral alterations and structural changes in hippocampal neuroglial networks, highlighting the importance of local vitamin D-dependent processes in maintaining brain integrity and function [48]. These observations raise the possibility that the cerebral requirements for vitamin D may differ from those of peripheral tissues and that currently accepted serum thresholds, which are largely based on skeletal outcomes, may not accurately reflect optimal conditions for neurological health. Furthermore, vitamin D influences multiple neurobiological pathways involved in neuroplasticity, neuroglial regulation, neurotransmission, and cognitive function. Emerging evidence also supports a role for vitamin D in the regulation of brain-derived neurotrophic factor (BDNF), a neurotrophin essential for neuronal survival, synaptic plasticity, learning, memory formation, and mood regulation [25,49]. Consequently, future investigations should move beyond conventional serum 25(OH)D concentrations and develop biomarker-based frameworks that better capture vitamin D’s broader neurobiological effects and its contribution to cognitive and emotional health [48,49].

Vitamin D deficiency in preclinical medical students: current data

Vitamin D deficiency is a common health concern among medical students worldwide. Although data from U.S. medical schools remain limited, studies from diverse international settings consistently report high rates of vitamin D insufficiency and deficiency, particularly during the preclinical years of training [50-52]. This trend is likely related to the nature of preclinical education, which involves prolonged indoor study, extensive classroom and laboratory attendance, reduced outdoor activity, irregular schedules, and limited exposure to UVB radiation, the primary source of endogenous vitamin D synthesis [8,51-54].

Evidence from multiple countries demonstrates the magnitude of the problem. A study evaluating 198 preclinical medical students reported vitamin D deficiency in nearly all participants, with prevalence rates of 92.6% among males and 99.0% among females. Importantly, low vitamin D levels persisted throughout the preclinical years, suggesting a sustained rather than temporary deficiency during medical training [8]. Similarly, another study found an overall deficiency prevalence of 74% among Libyan medical students aged 18-21 years. Only five male students and no female students met the criteria for vitamin D sufficiency, with differences attributed primarily to reduced sunlight exposure and clothing practices that limited cutaneous vitamin D synthesis [50]. Comparable findings have been reported in other regions. A cross-sectional study reported that 99% of medical students had serum vitamin D levels below 30 ng/mL, while 77% had levels below 20 ng/mL, indicating severe deficiency in most participants [51]. Collectively, these studies suggest that vitamin D deficiency among medical students is not restricted to a specific geographic location but represents a widespread global issue [8,50,51].

Lifestyle factors common among medical students appear to contribute substantially to this risk. Reduced outdoor activity, prolonged study hours, inadequate physical exercise, and extensive time spent indoors have all been associated with lower serum vitamin D concentrations [46,55]. These factors are especially relevant during the preclinical years, when academic demands frequently limit opportunities for sunlight exposure and physical activity. Consequently, the educational environment itself may function as an occupational risk factor for vitamin D deficiency. The broader occupational health literature supports this observation. In a systematic review of vitamin D status across occupations, Sowah et al. reported that predominantly indoor occupations were consistently associated with lower vitamin D levels than occupations involving regular outdoor exposure. Healthcare students and medical residents exhibited some of the lowest vitamin D concentrations, with deficiency rates of approximately 72% and 65%, respectively [52]. These findings suggest that healthcare education and training may predispose individuals to chronic vitamin D insufficiency.

The implications of vitamin D deficiency extend beyond skeletal health. Low vitamin D status has been associated with fatigue, musculoskeletal discomfort, reduced physical activity, impaired mood, and suboptimal cognitive performance, factors that may negatively affect academic functioning and overall well-being during medical training [46,53]. A recent review of healthcare workers further linked lower vitamin D levels with poorer health outcomes, reduced well-being, and decreased work productivity [56]. Given the substantial cognitive and psychological demands experienced by preclinical medical students, vitamin D deficiency may represent an underrecognized contributor to reduced quality of life and academic performance.

Overall, the available evidence indicates that preclinical medical students constitute a high-risk population for vitamin D deficiency. Although comprehensive studies in U.S. medical schools are lacking, the consistency of findings across international cohorts highlights the need for greater awareness, screening, and preventive strategies. Future research should focus on establishing prevalence rates among U.S. medical students and evaluating whether interventions such as education, lifestyle modification, and targeted supplementation can improve both health and academic outcomes [50-52,56].

Risk factors of vitamin D deficiency in preclinical medical students

Vitamin D status in preclinical medical students is influenced by a combination of biological, environmental, behavioral, and sociocultural factors. Although dietary intake and supplementation contribute to circulating 25(OH)D concentrations, cutaneous synthesis following UVB exposure remains the primary source of vitamin D for most individuals. Consequently, factors that reduce sunlight exposure or impair vitamin D synthesis significantly increase the risk of deficiency [52,57-59].

Sex-related and sociocultural differences appear to be important determinants of vitamin D status. A study reported a high prevalence of vitamin D deficiency among medical students in Pakistan despite adequate self-reported calcium intake and sun exposure, with female students exhibiting particularly low vitamin D levels [57]. Similarly, another study found substantially higher rates of deficiency among female medical students than males [50]. These findings have been attributed to factors such as clothing practices, reduced effective skin exposure to sunlight, and differences in outdoor activity patterns, highlighting the influence of both biological and behavioral factors on vitamin D status [50,57].

Lifestyle behaviors common among preclinical medical students are also major contributors to vitamin D deficiency. Reduced outdoor activity, low levels of physical exercise, and frequent use of sun-protective measures have been associated with lower serum vitamin D concentrations [58]. While sun protection is important for reducing ultraviolet-related skin damage, it may also limit UVB-mediated vitamin D synthesis. The demanding nature of preclinical medical education often requires prolonged indoor study, laboratory work, and classroom attendance, substantially reducing opportunities for regular sunlight exposure [50,58,59].

Additional lifestyle factors, including sedentary behavior, irregular schedules, and high academic workload, may further increase the risk of deficiency. Studies suggest that vitamin D insufficiency often develops through the cumulative effects of multiple behaviors common among medical students, including inadequate physical activity, prolonged indoor study, and limited outdoor recreation [53,54,57,59]. Thus, the preclinical learning environment itself may represent an important occupational risk factor for hypovitaminosis D.

Geographic location and ethnicity also influence vitamin D status. Higher latitudes are associated with reduced UVB exposure during portions of the year, limiting cutaneous vitamin D synthesis. In addition, increased skin melanin content decreases the efficiency of vitamin D production following sunlight exposure, placing some ethnic groups at greater risk for deficiency even under similar environmental conditions [54,58]. These factors may compound the effects of indoor lifestyles frequently observed among medical students. Evidence from occupational studies further supports the vulnerability of healthcare trainees. In a systematic review, Sowah et al. reported vitamin D deficiency rates of approximately 72% among medical students and 65% among medical residents, among the highest observed across occupational groups [52]. These findings underscore the significant impact of prolonged indoor activity associated with healthcare education and training.

Collectively, vitamin D deficiency in preclinical medical students results from the interaction of modifiable and nonmodifiable factors, including limited sunlight exposure, indoor academic schedules, low physical activity, sun-protective behaviors, sex, ethnicity, and geographic location. Strategies such as increased outdoor activity, lifestyle modification, dietary optimization, screening, and appropriate supplementation may help improve vitamin D status and support student health, psychological well-being, cognitive function, and academic performance [52,57-59].

Vitamin D deficiency and academic performance in preclinical medical students

Although studies specifically examining medical students remain limited, growing evidence suggests that vitamin D status may influence several factors that contribute to academic success. Higher serum 25(OH)D concentrations have been associated with better academic achievement and cognitive performance among student populations, indicating that adequate vitamin D status may support learning efficiency and educational outcomes [60,61]. Preclinical medical students may be particularly susceptible to the consequences of vitamin D deficiency because of the substantial cognitive load, prolonged study hours, and psychological stress associated with their training [46].

Low vitamin D concentrations have also been linked to increased symptoms of depression, anxiety, psychological distress, and cognitive fatigue, all of which can adversely affect concentration, motivation, memory retention, and examination performance [46,62,63]. These associations are particularly relevant in medical education, where sustained attention, rapid information processing, and effective memory consolidation are essential for academic success. Consequently, vitamin D deficiency may indirectly impair academic performance by reducing students’ capacity to cope with the intellectual and emotional demands of medical school [61-65]. Intervention studies provide additional support for this relationship. In a 10-week study of vitamin D-deficient university students, Dalibalta et al. reported significant improvements in working memory, verbal memory, and cognitive flexibility following vitamin D supplementation combined with physical activity [64]. These findings suggest that correcting vitamin D deficiency may enhance cognitive functions that are directly relevant to preclinical learning, including information retention, problem-solving, and executive functioning.

Despite increasing awareness of vitamin D’s importance, healthcare students often remain vulnerable to deficiency because of prolonged indoor study, limited sunlight exposure, and suboptimal sun-exposure practices [65]. Collectively, the available evidence suggests that vitamin D deficiency may negatively influence cognitive performance, psychological well-being, stress resilience, and academic achievement. Although causality has not been definitively established, maintaining adequate vitamin D status may represent a modifiable factor that supports both student well-being and academic performance during the demanding preclinical years of medical education [60-65].

Strategies to optimize vitamin D status in preclinical medical students

Given the high prevalence of vitamin D deficiency among medical students and its potential implications for physical health, psychological well-being, and academic performance, targeted preventive strategies should be incorporated into the preclinical learning environment [52,64]. Because many of the risk factors associated with vitamin D deficiency in medical students are lifestyle- and environment-related, interventions should focus on increasing awareness, improving sun exposure practices, promoting healthy behaviors, and facilitating appropriate supplementation when indicated [66-68].

Educational initiatives represent an important first step. Despite receiving health sciences training, many medical students demonstrate inadequate knowledge regarding vitamin D requirements, sources, and safe sun-exposure practices [64,66]. Incorporating brief educational modules on vitamin D health, nutrition, and lifestyle factors into student wellness programs may help improve awareness and encourage preventive behaviors [66]. Institutional support may also play a role in maintaining adequate vitamin D status. Given the prolonged indoor study schedules characteristic of preclinical training, medical schools could encourage regular outdoor breaks, provide access to outdoor study spaces, and promote physical activity programs that increase sunlight exposure. Such measures may help counteract the reduced UVB exposure associated with intensive academic schedules [52,67].

Nutritional interventions are equally important. Ensuring access to vitamin D-rich and vitamin D-fortified foods within campus dining facilities may help students meet recommended intake levels. For individuals at increased risk of deficiency or those with confirmed low serum 25(OH)D concentrations, targeted supplementation may be appropriate in accordance with established clinical guidelines [68]. Vitamin D supplementation has been shown to effectively improve vitamin D status and reduce the adverse consequences associated with deficiency [67,68]. An experimental model of vitamin D deficiency reported that vitamin D₃ supplementation improved behavioral and cognitive performance, including measures of spatial learning. The observed benefits were associated with neuroglial remodeling in the hippocampus, indicating that restoration of vitamin D status may support brain plasticity and improve neural mechanisms involved in learning and memory [48]. Promoting healthy lifestyle behaviors may provide additional benefits. Higher vitamin D concentrations are often associated with regular physical activity, outdoor recreation, and overall healthier lifestyle practices [52].

Furthermore, evidence suggests that interventions combining vitamin D optimization with physical activity may improve cognitive performance, memory, and psychological well-being in vitamin D-deficient university students [64]. Such findings support a comprehensive approach that integrates nutritional support, exercise, and lifestyle modification rather than relying solely on supplementation. Collectively, these strategies may help reduce the burden of vitamin D deficiency among preclinical medical students and support their physical health, cognitive function, and academic success throughout medical training [66-68].

## Conclusions

Vitamin D deficiency is highly prevalent among preclinical medical students and may adversely affect neuroendocrine function, stress resilience, cognitive performance, and immune health during a demanding stage of medical education. Evidence suggests that limited sunlight exposure, sedentary academic routines, and other modifiable lifestyle factors contribute substantially to this risk. Although current findings support a potential role for adequate vitamin D status in promoting well-being and academic performance, conclusions are limited by the predominance of observational studies, variability in vitamin D assessment methods, and insufficient data specific to U.S. medical students. Increased awareness, preventive strategies, and appropriately targeted interventions may help address this common deficiency, while future longitudinal and intervention studies are needed to clarify causal relationships and clinical benefits in medical trainees.
